# Cancer-Associated Stemness and Epithelial-to-Mesenchymal Transition Signatures Related to Breast Invasive Carcinoma Prognostic

**DOI:** 10.3390/cancers12103053

**Published:** 2020-10-20

**Authors:** Iulia-Monica Groza, Cornelia Braicu, Ancuta Jurj, Oana Zanoaga, Raduly Lajos, Paul Chiroi, Roxana Cojocneanu, Diana Paun, Alexandru Irimie, Schuyler S. Korban, Patriciu Achimas-Cadariu, Ioana Berindan-Neagoe

**Affiliations:** 1 11th Department of Oncology, Iuliu Hatieganu University of Medicine and Pharmacy, 400012 Cluj-Napoca, Romania; monica.groza@iocn.ro (I.-M.G.); airimie@umfcluj.ro (A.I.); 2 Department of Medical Oncology, The Oncology Institute “Prof. Dr. Ion Chiricuta”, 400015 Cluj-Napoca, Romania; 3 Research Center for Functional Genomics, Biomedicine and Translational Medicine, Iuliu Hatieganu University of Medicine and Pharmacy, 23 Marinescu Street, 400337 Cluj-Napoca, Romania; anca.jurj@umfcluj.ro (A.J.); oana.zanoaga@umfcluj.ro (O.Z.); lajos.raduly@umfcluj.ro (R.L.); paul.chiroi@stud.ubbcluj.ro (P.C.); roxana.cojocneanu@umfcluj.ro (R.C.); ioana.neagoe@umfcluj.ro (I.B.-N.); 4 Department of Endocrinology, Carol Davila University of Medicine of Pharmacy, 050474 Bucharest, Romania; diana.paun@umfcd.ro; 5 Department of Natural and Environmental Sciences, University of Illinois at Urbana-Champaign, Urbana, IL 61801, USA; korban@illinois.edu; 6 Department of Surgical Oncology and Gynecological Oncology, “Iuliu Hatieganu” University of Medicine and Pharmacy, 400000 Cluj-Napoca, Romania; 7 Department of Surgery, “Prof. Dr. Ion Chiricuta” Oncology Institute, 400015 Cluj-Napoca, Romania; 8 Department of Functional Genomics and Experimental Pathology, The Oncology Institute Prof. Dr. Ion Chiricuta, 34-36 Republicii Street, 400015 Cluj-Napoca, Romania

**Keywords:** breast cancer, mesenchymal transition, cancer stem cells

## Abstract

**Simple Summary:**

Breast cancer is one of the most common oncological diseases in women, as its incidence is rapidly growing. In this study, we have investigated the mechanism of epithelial-to-mesenchymal transition (EMT) and cancer stem cells (CSCs), demonstrating presence of an interconnectedness between them. This interconnectedness plays important roles in patient prognostic, as well as in diagnostic and therapeutic targets. It is identified that there is a common signature between CSCs and EMT, and this is represented by ALDH1A1, SFRP1, miR-139, miR-21, and miR-200c. This finding will provide a better understanding of this mechanism, and will facilitate the development of novel treatment options.

**Abstract:**

Breast cancer is one of the most common oncological diseases in women, as its incidence is rapidly growing, rendering it unpredictable and causing more harm than ever before on an annual basis. Alterations of coding and noncoding genes are related to tumorigenesis and breast cancer progression. In this study, several key genes associated with epithelial-to-mesenchymal transition (EMT) and cancer stem cell (CSC) features were identified. EMT and CSCs are two key mechanisms responsible for self-renewal, differentiation, and self-protection, thus contributing to drug resistance. Therefore, understanding of the relationship between these processes may identify a therapeutic vulnerability that can be further exploited in clinical practice, and evaluate its correlation with overall survival rate. To determine expression levels of altered coding and noncoding genes, The Cancer Omics Atlas (TCOA) are used, and these data are overlapped with a list of CSCs and EMT-specific genes downloaded from NCBI. As a result, it is observed that CSCs are reciprocally related to EMT, thus identifying common signatures that allow for predicting the overall survival for breast cancer genes (BRCA). In fact, common CSCs and EMT signatures, represented by ALDH1A1, SFRP1, miR-139, miR-21, and miR-200c, are deemed useful as prognostic biomarkers for BRCA. Therefore, by mapping changes in gene expression across CSCs and EMT, suggesting a cross-talk between these two processes, we have been able to identify either the most common or specific genes or miRNA markers associated with overall survival rate. Thus, a better understanding of these mechanisms will lead to more effective treatment options.

## 1. Introduction

Breast cancer is a high-frequency disease due to its increased incidence, as recorded for the past few years, and it is now ranked as the second-most deadly form of malignancy in women after lung cancer [1]. The most common subtype is represented by invasive breast cancer. Despite promising progress made in pursuing combined treatment strategies, such as surgery, radiation, and chemotherapy, there remains a significant number of cases where these procedures are incapable of preventing the occurrence of metastatic disease [2,3]. Therefore, patient care management remains an important clinical challenge due to the lack of availability of prognostic markers that could stratify a patient’s risk. In the past few years, there has been an increased interest in verifying the clinical utility of coding and noncoding genes as important biomarkers for prognostic, diagnostic, and therapeutic targets [2,4,5,6,7,8].

Solid tumors, including breast cancer-associated tumors, comprise a particular subpopulation of tumor cells, referred to as distinct cancer stem cells (CSCs). CSCs have an infinitely proliferative potential, multipotency, and self-renewal capacity that could altogether confer resistance to antitumoral treatments [9], as well as facilitate tumor recurrence and metastasis [10,11].

It has been observed in multiple cases that CSCs display comparable phenotypes to those of normal stem cells (SCs). Furthermore, as it is already well-known, SCs play a key role in both embryogenesis and adult/mature organisms, wherein SCs are confirmed to be involved in various diverse regenerative processes [12]. The most common shared feature of these two types of stem cell populations is related to their capacity for self-renewal. This observed high potential for cell proliferation is linked with resistance to replicative senescence [12], thus rendering these CSCs capable of driving tumor growth, progression, and metastasis due to the expression of stem cell-like features, including CD44/CD24 and ALDH1 [13]. Furthermore, Wnt (Wingless-related integration site)/β-catenin, Hedgehog, and Notch are reported to serve as shared signaling pathways [12].

CSCs serve as a considerable challenge when they are intended for use in fighting cancer. CSCs sustain self-renewing tumorigenic cell fractions; moreover, these cells are involved in drug resistance, and they are linked to invasion and distant metastasis [14]. Compared to non-stem cancer cells, CSC populations possess higher levels of resistance to chemotherapy, radiotherapy, and immunotherapy [5,14].

Epithelial-to-mesenchymal transition (EMT) represents a biological program during which epithelial cells lose their identity and acquire a mesenchymal phenotype [15]. Thus, EMT has dual biological roles, playing a physiological role in sustaining organismal development, but under pathological conditions such as cancer, EMT is associated with increased invasion and migration rates that further promote high metastatic rates [15,16]. This process can be snatched by cancer cells, being frequently linked with resistance to apoptosis, acquisition of cancer stem cell characteristics, and drug resistance [16]. EMT programs/processes/mechanisms promote CSC stemness in many epithelial tissues; therefore, understanding these relationships may highlight different therapeutic vulnerabilities that can be further exploited in clinical practice [17].

It has been often reported that breast cancer has CSC signatures at the functional, molecular, and transcriptional levels [10,18]. Recent studies have found that EMT and CSCs are likely to be associated with a high risk for breast cancer recurrence and poor prognosis. Hence, understanding the mechanisms of breast cancer pathogenesis will aid in the discovery of effective treatment options [19,20].

The Cancer Omics Atlas (TCOA, http://tcoa.cpu.edu.cn) was established to allow for fast and straightforward querying of the TCGA “omics” data [21]. These data will allow for investigating transcriptomic patterns of gene expression of population(s) of CSCs, development and maintenance of CSC phenotype(s), and regulation modes of the cytoprotective mechanism involved in cell survival, thus evading attack by the immune system. These investigations will contribute to a better understanding of CSCs and their interconnectedness with EMT biology, as this leads to identifying major determinants of breast cancer biology.

In an earlier study, we observed critical transcriptomic alterations in breast cancer tissues [5]. Among these, miR-200c and the critical regulators of EMT and CSCs are likely to be useful in the diagnostic/prognostic of her2-positive breast cancer [5]. Therefore, pursuing additional investigations of those common EMT and CSCs signatures will have important clinical benefits in breast cancer management, as these two processes are mediators of resistance to therapy [22]. Therefore, the findings obtained in this study will improve the development of novel therapeutic agents, as well as aid in undertaking efforts for developing enhanced and more effective clinical practices for the management of breast cancer.

## 2. Results

### 2.1. Breast Cancer Cell Analysis Reveals Distinct Expression Profiles for EMT

Gene expression analysis using TCOA revealed the presence of 2368 altered genes, consisting of 674 overexpressed and 1694 downregulated genes, at a cut-off value |Fold change| > 1.5 and False Discovery Rate (FDR) q-value ≤ 0.05 (Table S1). Moreover, miRNA analysis identified altered expression patterns for 47 transcripts, consisting of 19 overexpressed and 28 downregulated miRNAs (Table S2). Those most frequent changes are displayed in Table S3. In addition, a list of the top 10 upregulated and downregulated genes, along with their respective miRNAs, are presented in Table 1.

### 2.2. Integrating Altered Genes to Tumorigenesis and Molecular Pathways

**Figure 1:**
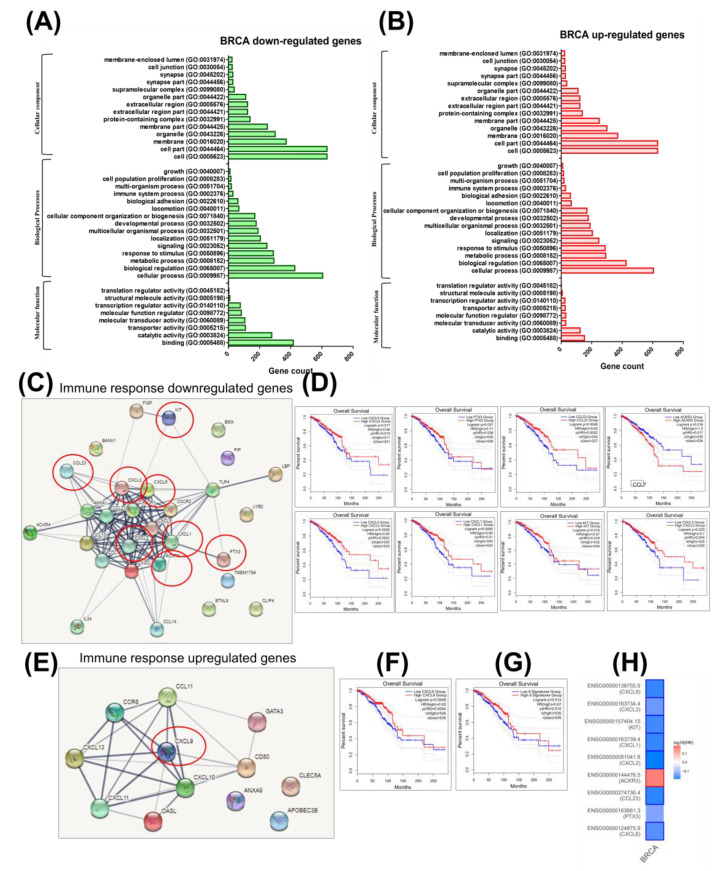
Biological significance of the breast cancer (BRCA)-altered gene expression signatures. (**A**) Gene ontology (GO) analysis for downregulated genes and (**B**) GO analysis for overexpressed/upregulated genes, respectively, using the Panther Gene ontology tool (http://pantherdb.org). (**C**) A BRCA immune response functional interaction network based on downregulated genes generated using STRING 11.0 software. (**D**) Overall survival rate for the downregulated immune response BRCA signature (CXCL6, PTX3, CCL23, ACKR3, CXCL2, CXCL1, KIT, and CXCL3) of genes with *p* ≤ 0.05 generated using GEPIA2. (**E**) A BRCA immune response and a functional interaction network based on overexpressed genes. (**F**) Overall survival rate for a single overexpressed (CXCL9) gene. (**G**) An overall survival rate for a 13-gene signature (CXCL6, PTX3, CCL23, ACKR3, CXCL2, CXCL1, KIT, CXCL3, and CXCL9), and (**H**) a survival map for these selected 9 gene signatures.

Gene Ontology (GO) for all 2368 altered genes was conducted using the web-based software Panther (http://www.pantherdb.org). Functions of both upregulated and downregulated mRNAs (tumoral versus normal tissues) were assigned. The main biological functions altered were related to catalytic activity for both up/downregulated genes. The classification of related molecular functions revealed alterations of cellular processes and biological regulation (Figure 1A,B).

The panel of genes responsible for immune response regulation (based on Panther Gene Ontology classification) points to 29 overexpressed genes, and these are displayed as a network in Figure 1C. GO is used to further validate involvement of these signature genes in CSC and EMT pathways in breast cancer. Of these, eight genes, including CXCL6, PTX3, CCL23, ACKR3, CXCL2, CXCL1, KIT, and CXCL3, are correlated with the overall survival, and these are displayed in Figure 1D. Moreover, a String network for downregulated genes is presented in Figure 1E, and among these, a single gene, CXCL9, is found to be correlated with the overall survival rate (Figure 1F–H).

### 2.3. Construction of a Gene Network Involved in CSCs and EMT for Breast Cancer Genes (BRCA)

CSCs and EMT are two key mechanisms involved in several solid tumors, including those for breast cancer, and they are responsible for self-renewal, differentiation, and self-protection, as well as contributing to drug resistance. When lists of specific transcripts for CSC and EMT are downloaded from NCBI and overlapped with the altered genes, a group of common downregulated and upregulated genes is identified.

**Figure 2:**
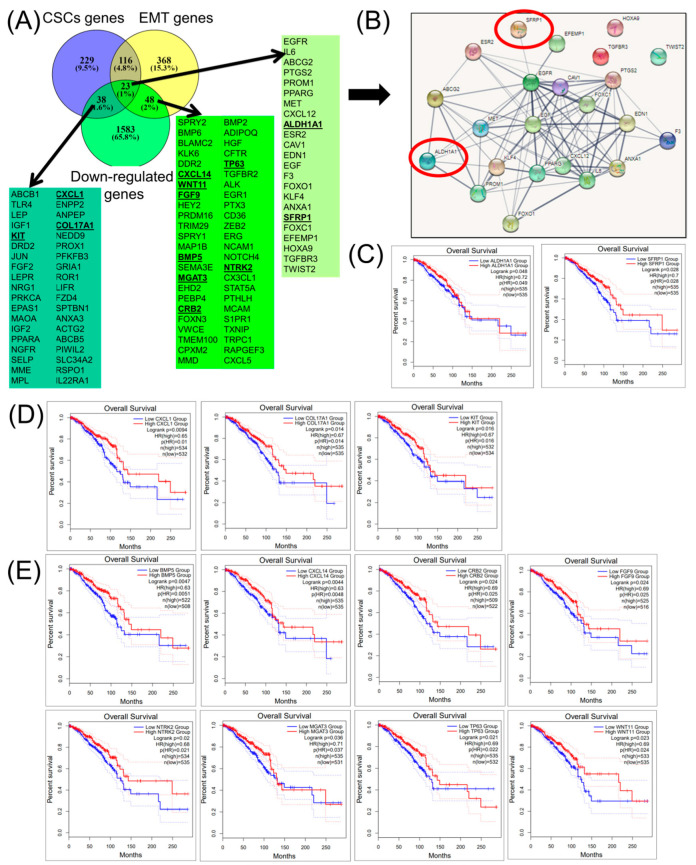

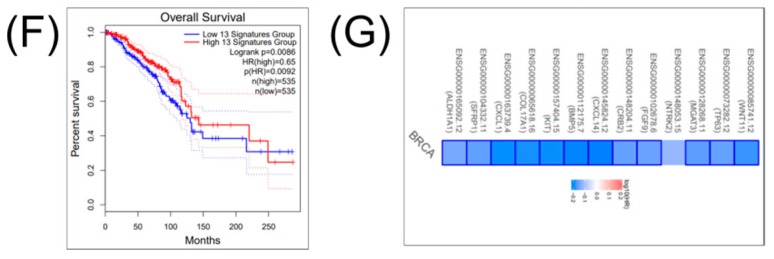
Common cancer stem cells (CSCs) and epithelial-to-mesenchymal transition (EMT) of a downregulated gene expression signature in BRCA. (**A**) A Venn diagram presenting downregulated genes for CSCs and EMT in BRCA (downloaded from NCBI), as well as common signature genes for both CSC and EMT gene lists; bold-lettered genes predict the overall survival. (**B**) An interaction network using String software for a 23-common gene signature involved in both CSC and EMT mechanisms; red-circled genes predict the overall survival outcomes. (**C**) BRCA-altered genes involved in both CSCs and EMT (ALDH1A1 and SFRP1) predicting the overall survival. (**D**) BRCA-altered genes involved only in CSCs (CXCL1, COL17A1, and KIT). (**E**) BRCA-altered genes involved only in EMT (BMP5, CXCL14, CRB2, FGF9, NTRK2, MGAT3, TP63, and WNT11) predicting the overall survival. (**F**) Overall survival rate of a 13-gene signature (ALDH1A1, SFRP1, CXCL1, COL17A1, KIT, BMP5, CXCL14, CRB2, FGF9, NTRK2, MGAT3, TP63, and WNT11). (**G**) A survival map of the selected 13-gene signature.

Of downregulated genes, 23 genes display a common shared signature for both CSCs and EMT gene lists (Figure 2A), and this is presented as a network (Figure 2B). Of these, only ALDH1A1 and SFRP1 are statistically significant, and are correlated with the overall survival rate (Figure 2C). Moreover, of CSC-specific genes, 38 genes are found in common, but only three genes (CXCL1, COL17A1, and KIT) are correlated with the overall survival rate (Figure 2D). In addition, from a panel of 48 EMT-associated genes, only eight genes (BMP5, CXCL14, CRB2, FGF9, NTRK2, MGAT3, TP63, and WNT11) are correlated with the overall survival rate, and these are displayed in Figure 2E. In Figure 2F, the overall survival of the 13-gene signature (ALDH1A1, SFRP1, CXCL1, COL17A1, KIT, BMP5, CXCL14, CRB2, FGF9, NTRK2, MGAT3, TP63, and WNT11) is presented; meanwhile, in Figure 2G, a graphical representation of same gene signature as the survival map is presented.

**Figure 3:**
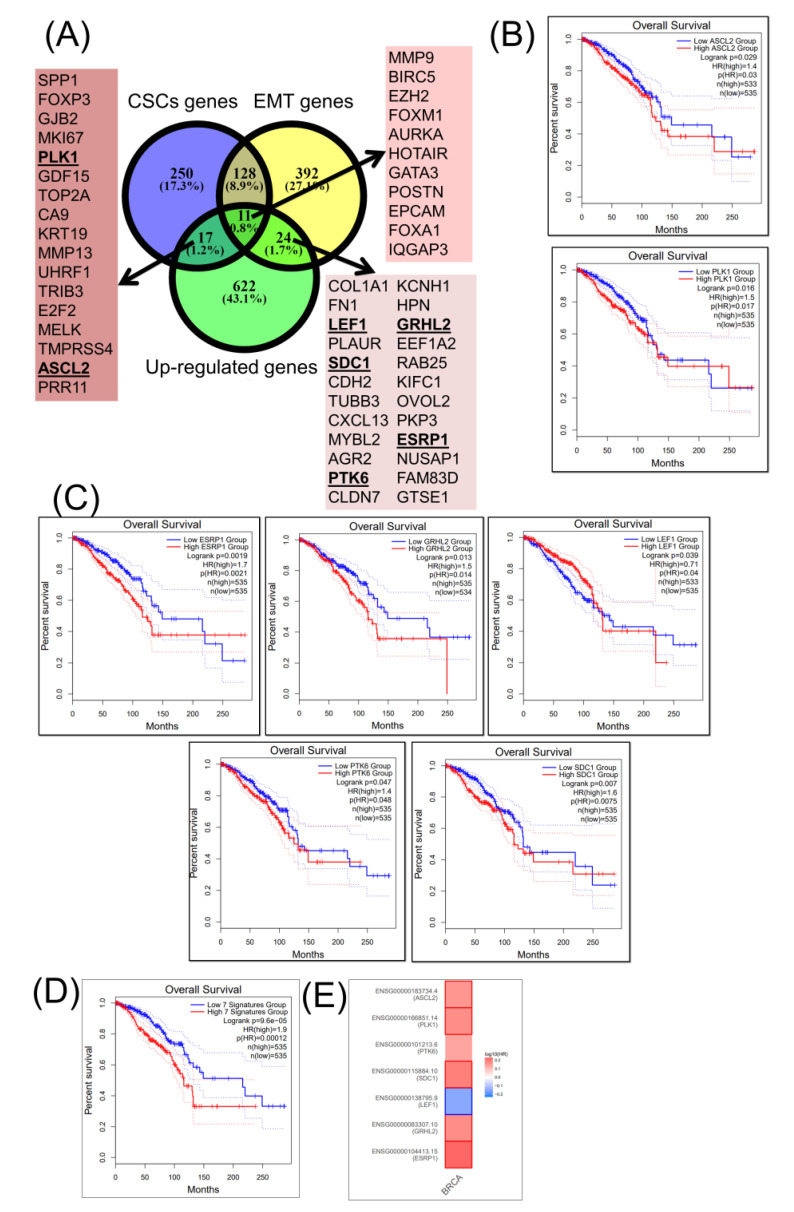
A common CSC and EMT upregulated (overexpressed) gene expression signature in BRCA. (**A**) A Venn diagram presenting altered upregulated genes of CSCs and EMT in BRCA (downloaded from NCBI), as well as a common signature among CSC and EMT gene lists; bold-lettered genes predict the overall survival. (**B**) BRCA-altered genes involved only in CSCs (PLK1 and ASCL2) predicting the overall survival. (**C**) BRCA-altered genes involved only in EMT (ESRP1, GRHL2, LEF1, SDC1, and PTK6) predicting the overall survival. (**D**) Overall survival rate of a seven-gene signature (ESRP1, GRHL2, LEF1, SDC1, PTK6, PLK1, and ASCL2). (**E**) A survival map of the selected seven-gene signature.

Of the upregulated genes, 11 genes (MMP9, BIRC5, EZH2, FOXM1, AURKA, HOTAIR, GATA3, POSTN, EPCAM, FOXA1, and IQGAP3) display a common shared signature for both CSCs and EMT gene lists (Figure 3A), but none are correlated with the overall survival rate (Figure 3B). Moreover, of the 24 genes associated with EMT (Figure 3C), only five genes (ESRP, GRLHL2, LEF1, SDC1, and PTK6) are correlated with the overall survival rate (Figure 3D); whereas, of CSC-associated genes, only PLK1 and ASCL2 are statistically significant. In Figure 3D, the overall survival of the seven-gene signature (ESRP1, GRHL2, LEF1, SDC1, PTK6, PLK1, and ASCL2) is presented; meanwhile, in Figure 3E, a heatmap representation for the same gene signature is presented.

### 2.4. Construction of a miRNA Network Involved in both CSCs and EMT

**Figure 4:**
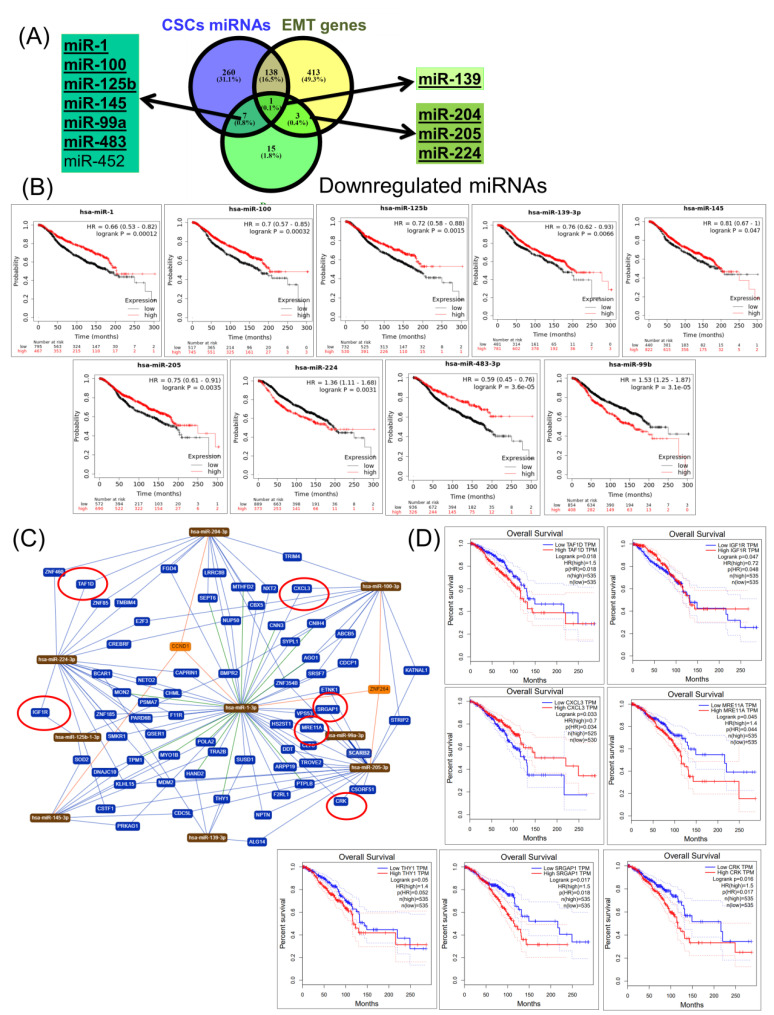

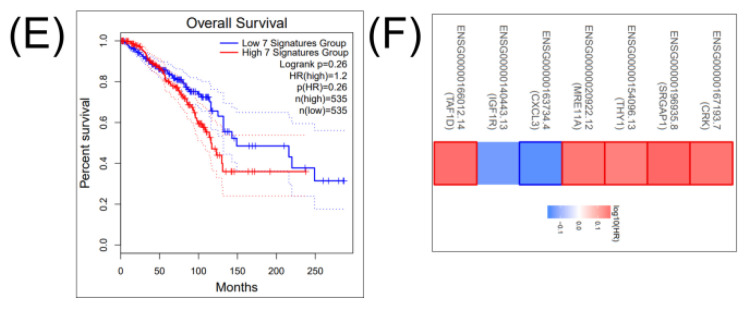
A common CSC and EMT downregulated miRNA expression signature in BRCA. (**A**) A Venn diagram of the downregulated genes of CSCs and EMTs in BRCA (downloaded from NCBI), as well as a signature of common miRNAs for both CSC and EMT transcript lists; bold-lettered genes predict the overall survival. (**B**) miRNAs capable of predicting the overall survival (miR-1, miR-100, miR-125b, miR-139, miR-145, miR-205, miR-224, miR-483, and miR-99b). (**C**) An interaction network using miRTargetLink software (strong interaction) for miRNAs involved in both CSCs and EMT and correlated with the overall survival. (**D**) An overall survival analysis for genes targeted by miRNA and correlated with the survival rate (TAF1D, IGF1R, CXCL3, MRE11A, THY1, SRGAP1, and CRK). (**E**) Overall survival rate of a seven-gene signature (TAF1D, IGF1R, CXCL3, MRE11A, THY1, SRGAP1, and CRK). (**F**) A survival map of the seven-gene signature.

**Figure 5:**
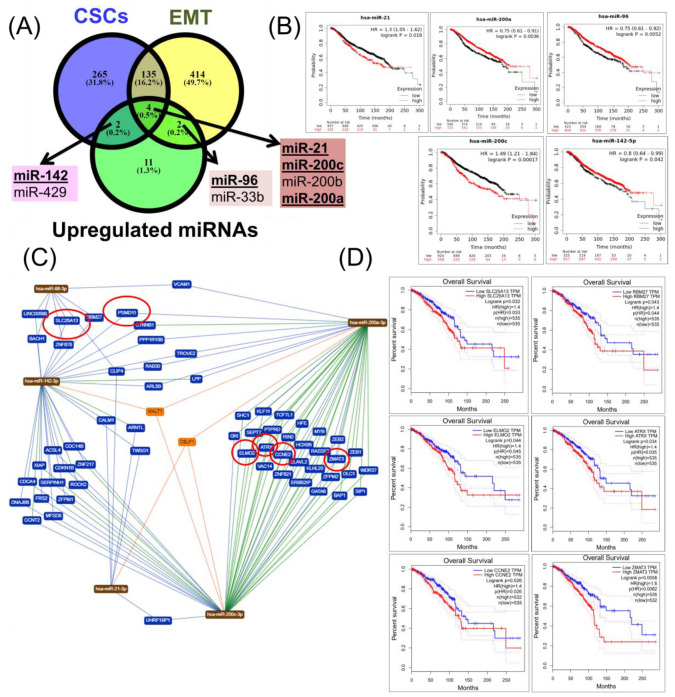

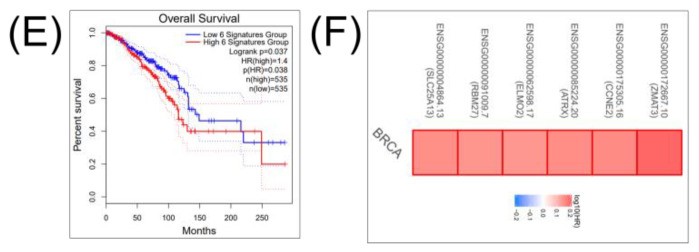
A common CSC and EMT overexpressed miRNA expression signature in BRCA. (**A**) A Venn diagram of the downregulated miRNAs of CSCs and EMT in BRCA (downloaded from NCBI), as well as a common signature among these CSC and EMT transcript lists; bold-lettered genes predict the overall survival. (**B**) Those miRNAs capable of predicting the overall survival (miR-21, miR-96, miR-142, miR-200a, and miR-200c). (**C**) An interaction network using miRTargetLink software (strong interaction) for miRNAs involved in CSCs and EMT and correlated with the overall survival. (**D**) Overall survival analysis of genes targeted by miRNA and correlated with the survival rate (SLC25A13, RBM27, ELMO2, ATRX, CCNE2, and ZMAT3). (**E**) Overall survival rate of a six-gene signature (SLC25A13, RBM27, ELMO2, ATRX, CCNE2, and ZMAT3). (**F**) A survival map of the selected six-gene signature.

For downregulated miRNAs, it was observed that miR-139 was common in both CSC and EMT transcript lists (Figure 4A). Moreover, three miRNAs (miR-204, miR-205, and miR-224) were specific for EMT, while seven miRNAs (miR-1, miR-99a, miR-100, miR-125b, miR-145, miR-452, and miR-483) were specific for CSCs (Figure 4A). Except for miR-452, all common transcripts were correlated with the overall survival rate (Figure 4B). Using miRTargetLink, miRNAs were identified that are most relevant to the target genes (Figure 4C,D), but only 11 miRNAs (TAF1D, CRK, SRGAP1, RANGAP1, KDELR1, PRKG2, SEC63, DHX33, OSBPL10, CXCL3, and IGF1R) were found to be capable of predicting the overall survival (Figure 4D). The overall survival rate of a seven-gene signature (TAF1D, IGF1R, CXCL3, MRE11A, THY1, SRGAP1, and CRK), along with a survival map of the seven-gene signature, are presented in Figure 4E,F. For upregulated miRNAs, it was observed that there were four common transcripts (miR-21, miR-200a, miR-200b, and miR-200c) between the CSC and EMT transcript lists, and of these, only three miRNAs (miR-21, miR-200a, and miR-200c) were correlated with the overall survival (Figure 5A,B). Moreover, it was observed that there were two miRNAs that were specific for EMT, but only a single miRNA, miR-96, that predicted the overall survival (Figure 5A,B). In addition, there were two miRNAs that were specific for CSCs but only a single miRNA, miR-142, that predicted the overall survival (Figure 5A,B). Using miRTargetLink, miRNAs were identified that were the most relevant to the target genes (Figure 5C,D), but only six (SLC25A13, RBM27, ELMO2, ATRX, CCNE2, and ZMAT3) were capable of predicting the overall survival in BRCA (Figure 5D). Furthermore, the overall survival rate of a six-gene signature (SLC25A13, RBM27, ELMO2, ATRX, CCNE2, and ZMAT3), along with the graphical representation and survival map of the selected six-gene signature, are presented in Figure 5E,F.

### 2.5. Validation of Altered Transcriptomic Patterns by Quantitative Real-Time Polymerase Chain Reaction (qRT-PCR)

**Figure 6:**
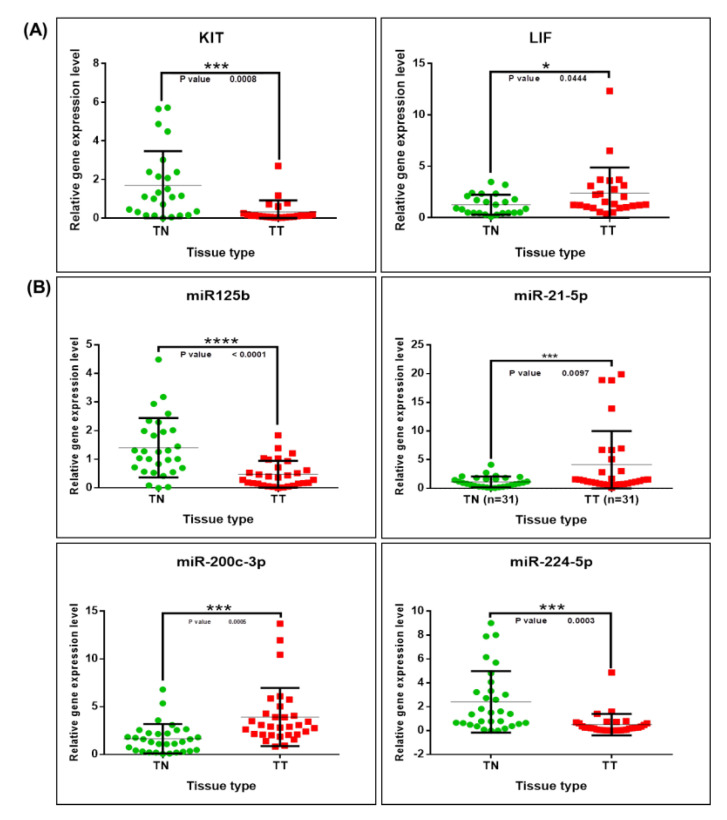
Gene and miRNA expression alterations in breast cancer. (**A**) Scatter plots demonstrating the downregulation of KIT and upregulation of LIF in tumor tissues versus normal tissues. (**B**) Scatter plots showing the downregulation of miR-125b and miR-224-5p, as well as the upregulation of miR-21-5p and miR-200c-3p in tumor tissues versus normal tissues. For normalization of the gene expression data, B2M and GAPDH were used as the internal controls, whereas, for miRNA data, U6 and RNU48 were used as the internal controls, based on the ΔΔCt method (* *p* ≤ 0.05, *** *p* ≤ 0.001, **** *p* ≤ 0.0001).

In order to further validate the observed gene and miRNA expression changes, qRT-PCR was conducted for KIT and LIF genes, wherein B2M and GAPDH genes were used as endogenous controls for normalization of the qRT-PCR data. Validation of these genes and miRNA transcripts were conducted using 30 tissue samples collected from the early stages of breast cancer, as well as of matched pairs of 30 samples of distant normal tissues. A gene expression analysis revealed that KIT levels were significantly lower, while those of LIF were significantly higher (overexpressed), in tumor tissues, compared to those of normal tissues (Figure 6). For miRNA analysis, both miR-125b and miR-224-5p levels were lower, while those for both miR-21-5p and miR-200c-3p were higher in tumor tissues versus normal tissues (Figure 6).

These qRT-PCR results further validate our earlier gene and miRNA expression profiles. Furthermore, these findings suggest that, in the early stages of breast cancer, these genes and miRNAs are useful as biomarkers and as therapeutic targets.

## 3. Discussion

Despite recent advances and significant progress made in cancer treatment, acquired resistance to chemotherapeutics remains a major barrier to effectively treat patients, affecting the overall medical outcome. Therefore, exploring major determinants of the breast cancer biology would be helpful for improving diagnosis and treatment of breast cancer, and will also allow for identification of novel therapeutic targets. As there are several molecular pathways exploited by breast cancer cells with EMT, along with those residing in a stem-like state [22], these mechanisms were also confirmed in our study. In particular, it was observed that CSCs are closely related to EMT, and that EMT is likely to be critical in tumor invasion and metastasis [17,23]. Furthermore, CSCs are responsible for tumorigenesis-associated processes, as these cells display increased resistance to therapy, even promoting tumors post-treatment relapse [17]. Thus, identification of transcriptional markers for these two pathways in order to understand resistance towards some of the most prevalently used therapies for treatment of breast cancer will greatly benefit breast cancer patients. In addition, this will allow for identification of novel targeted therapies, and also for prognostic markers for disease recurrence. In this study, the overall findings confirm the critical roles of key EMT and CSC genes in breast cancer patient prognostics.

It is known that cytokines, chemokines, and inflammatory mediators released by tumor microenvironment components influence proliferation, tumorigenic transformation, and/or apoptosis of CSCs by various signaling pathways [20]. It was demonstrated that cytokines and chemokines are overexpressed in aggressive BRCA tumors, and CXCL2, CXCL3, CXCL5, CXCL6, and CXCL8 chemokines are present at higher levels in metastatic cases [24]. Mechanistic analyses revealed that the CXCL16/CXCR6 chemokine axis is responsible for regulation of the invasiveness and metastasis of BRCA via activation of the ERK1/2 signaling pathway [25]. It was observed that PARP inhibitors related with ERK inhibition reduce the expression level of proangiogenic factors like the case of vascular endothelial growth factor (VEGF) and hypoxia inducible factor (HIF) [26], supporting the utility of CXCL16/CXCR6 not only as biomarker but, also, as a therapeutic target.

A panel of five genes (CXCL12, IGF1, LEF1, MMP1, and RACGAP1) was proposed as biomarkers for prognosis through the survival analysis of BRCA [27]. MMPs are rather considered as target genes of EMT pathways and MMP expression as a late event of the EMT, being interconnected with key transcription factors, such as Snail, ETS, and β-catenin [28,29].

In another study, a signature of four immune-related genes (APOD, CXCL14, IL33, and LIFR) was correlated with breast cancer prognosis [30]. In our study, it was revealed that these EMT markers are also associated with overall survival rates. Furthermore, an atypical chemokine receptor 3 (ACKR3) is proposed as a therapeutic target [31], as it is directly related to CXCR4 and CXCL12 [31], thereby promoting cellular migration and activation of the ERK and Akt pathways [31]. It has been observed that Notch signaling regulates the expression of SEMA3C, CXCL14, CCL20, CXCR7, and HMGA2, and these are proposed as markers for prometastatic processes [32]. In a recent study, it was reported that PTX3, SNAI2, IL-8/6, SPARC, MMP-1, and Rab25 are key therapeutic targets in metastatic breast cancer [33]. PTX3, a key/critical element for PI3K-induced stem cell-like traits and for EMT, is associated with poor survival rates via PI3K [34,35]. This observation was further confirmed by the findings obtained in our study.

It is known that CSCs efficiently express ATP-binding cassette (ABC) transporters, deemed as multidrug resistance proteins, and are capable of protecting cells from drug damage, and they are also involved in inducing drug resistance [23,36]. Aldehyde dehydrogenase (ALDH), a marker for CSCs and EMT, is recognized as having a capability in eliminating oxidative stress, and in increasing resistance to chemotherapeutic drugs [37]. In our study, it is observed that the aldehyde dehydrogenase family 1 member A1 (ALDH1A1) is correlated with overall survival in BRCA. Previously, it is reported that overexpression of ALDH1A1, an isozyme linked to CSCs and EMT, is related to different poor prognostic outcomes [38,39,40]. Furthermore, a secreted frizzled-related protein 1 (SFRP1) is known to be responsible for hyperplasia [41], and thus, lack of SFRP1 is accompanied by both tumor development and poor prognosis for breast cancer [41]. In our study, we found that both ALDH1A1 and SFRP1, two commonly important genes for EMT and CSCs, are capable of predicting the overall survival rates for breast cancer.

In this study, we identified yet another CSC marker; COL17A1, a novel TP53 target [42] and a frequently mutating gene in breast cancer (Table S3), is known to be involved in the regulation of both cell migration and invasion [42,43]. Moreover, additional markers identified include EGFR, AP-1, p63, and TGF-β, as their pro-oncogenic functions regulate breast cancer invasiveness, and therefore, these can be exploited as therapeutic targets in breast cancer [44]. The p53 family member p63 is a transcriptional regulator of epithelial development and differentiation; moreover, p63 is also involved in the transcriptional regulation of EGFR genes [44], frequently mutating genes in breast cancer (Table S3). In addition, in this study, we identified TP63, an EMT gene, as a member of a 13-gene signature for overall survival. TP63 isomorphs are reported to be related to different basal phenotypes [45]. Furthermore, it is known that TP63 regulation via PI3K/Akt and immune response markers promote drug resistance in breast cancer [46].

It has been reported that TGF-β1 induced EMT via repression of BMP5 in breast cancer [47]. SDC1, another EMT marker identified in this study, can be used to assess the tumor prognosis [48], including breast cancer, wherein increased expression levels of SDC1 are associated with the worst prognosis [49]. This observation is confirmed by findings obtained in our study. Moreover, it has been demonstrated in a previous study that SCD1 can also promote brain metastasis [50].

In this study, lower levels of the expression of miR-96-5p in CSCs and EMT are observed. Apparently, this is a common characteristic of many cancer types, including breast-related cancers. MiR-96-5p targets and downregulates catenin delta 1 (CTNND1), leading to decreased expression of β-catenin [51] and loss of WNT11 signaling, thus reducing the cyclin D1 levels and MMP7 expression [51].

Yet another transcript, miR-21, detected in the common signature of CSCs and EMT in our study, is found to be correlated with the overall survival. It has been reported that miR-21 is generally overexpressed in most solid tumors, and in breast cancer, this oncomiR is associated with lymph node metastasis, clinical staging, and differentiation [52]. The utility of serum miR-21 has been intensively investigated as a marker of colorectal cancer (CRC) diagnosis and progression [53]. In stage II CRC patients, a high expression of miR-21 is associated with shorter PFS. It also represents a novel predictive marker for the recurrence of stage II CRC [54]. In addition, miR-21 can influence the response to chemotherapy, triggering an IL-6/STAT3/NF-κB-mediated signaling loop, along with activation of PI3K signaling [55]. Moreover, the inhibition of miR-21 is correlated with the inhibition of cell migration and invasion by blocking PI3K/AKT signaling pathways and reversing the EMT [56].

It has been reported that TP53 regulates EMT and CSCs by modulating miRNAs [57]. Therefore, the observed loss of TP53 in our study leads to lower levels of miR-200c, thereby promoting expression of EMT and CSCs markers [57]. In another study, it was reported that miR-200c, resulting from a mutation in p53, can upregulate the Moesin oncogene, thereby promoting carcinogenesis [58]. In fact, MiR-200c regulates EMT by inhibiting ZEB1 and ZEB2 expression in breast cancer cells [59], while it regulates CSCs heterogeneity via targeting the HIPK1/β-Catenin axis [60].

**Figure 7:**
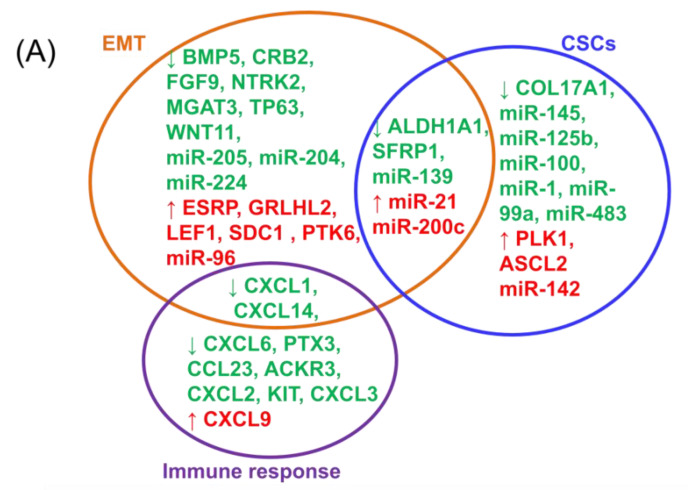

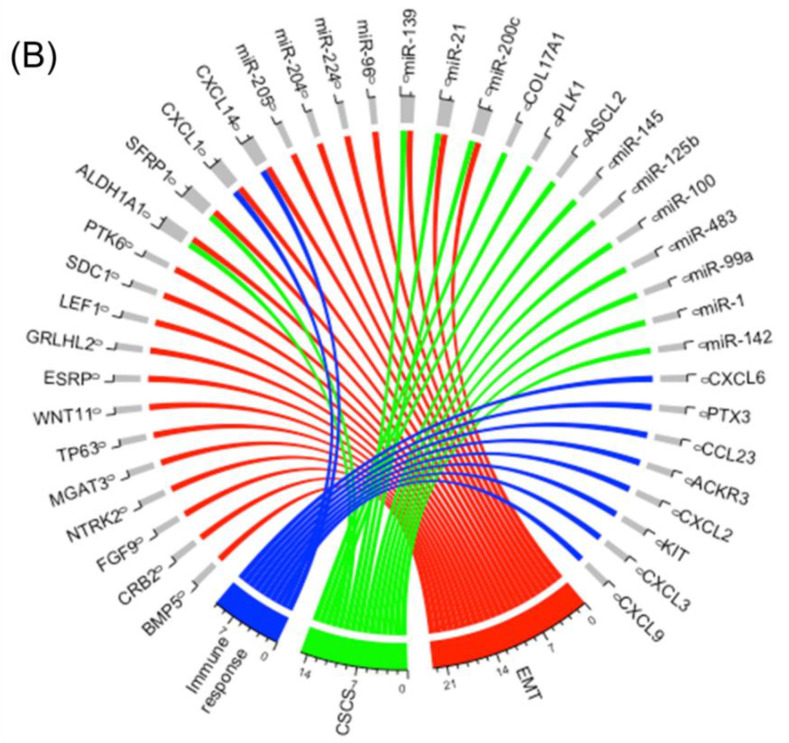
Common and specific transcripts of EMT and CSCs related to the BRCA overall survival. (**A**) A Venn diagram of EMT and CSC transcripts predicting the overall survival, and (**B**) a Circos representation of transcripts predicting the overall survival rate involved in the immune response, CSCs, and EMT.

Although various gene expression studies, along with additional information, have already been previously conducted, there have not been any detailed analyses to identify gene and transcript signatures that can be used for prognostics for overall survival. In this study, both common and specific genes and transcripts of CSCs and EMT for BRCA were identified (Figure 7). Furthermore, validation of early stage breast cancer confirms the critical role of common EMT and CSCs signatures for a patient prognostic (Figure 7). These common signatures identified in this study will have important roles as prognostics for overall survival. This is of particular importance for patients for whom clinical parameters and traditional immunohistochemical markers would lead to an unequivocal prognosis [61].

## 4. Materials and Methods

### 4.1. Differential Gene and miRNA Expression Analysis for BRCA

Expression data have been retrieved from the TCOA, an important database for exploring TCGA records. This resource does not require high-level bioinformatics expertise, thus allowing the user to run different types of analyses. In the “Cancer” module, a user is allowed to select particular cancer types, and TCOA will further output the top 50 most frequently mutated genes, both upregulated and downregulated genes, as well as upregulated and downregulated miRNAs. All these values are provided in association with selected pathologies and, also, compared to normal controls.

### 4.2. Network Analysis

In order to predict network interactions that will further help in either developing or discovery of likely biomarkers for clinical diagnosis, or in highlighting novel therapeutic targets for BRCA, the bioinformatic tool String version 11.0 (https://string-db.org) was used in this study. A gene enrichment analysis was performed using PANTHER Gene Expression Analysis Tools, a software providing distinct classifications of the molecular function, biological process, or pathway associations of selected data [62].

### 4.3. Survival Analysis

For evaluations of both gene expression data and their correlations with breast cancer survival rates based on TCGA and GTEx databases, a GEPIA online tool was used (http://gepia.cancer-pku.cn/). A miRNA survival analysis for BRCA was evaluated using miRpower based on a sample dataset of *n* = 1262 breast cancer patients (METABRIC) (http://kmplot.com/analysis/index.php?p=service&default=true) [63]. Only those genes capable of predicting overall survival outcomes (*p*-value ≤ 0.05) were presented.

### 4.4. Gene and miRNA Validation Using Early Stage Breast Cancer Tissue Samples

A total of 30 histologically confirmed breast cancer patients admitted at The Oncology Institute “Prof. Dr. Ion Chiricuta” Cluj-Napoca, Romania during 2018–2020 were enrolled in this study. The study was approved by the institutional ethical committees, and informed consent was collected from all patients. The age of patients ranged between 33–76 years. All patients were staged according to the American Joint Committee on Cancer (AJCC) guidelines. Expression of the estrogen receptor (ER), progesterone receptor (PR), and human epidermal growth factor receptor 2 (HER2/neu) was conducted using immunohistochemistry (IHC). Immediately following a modified radical mastectomy or following an incision biopsy, all tissue samples were snap-frozen in liquid nitrogen for RNA isolation and stored at −80 °C. Stages I and II patients were selected; most were ER- and PR-positive, as described in Table 2.

Total RNA extraction was performed using TriReagent (Ambion, Austin, TX, USA), according to manufacturer’s instructions. Then, total RNA quality and quantity were evaluated using a NanoDrop 2000 spectrophotometer (Thermo Scientific, Waltham, MA, USA), and 1000 ng of total RNA was reverse-transcribed into cDNA using a High-Capacity cDNA Reverse Transcription Kit (Applied Biosystems, Foster City, CA, USA). Subsequently, gene expression levels were determined using a SYBR Select Master Mix (Applied Biosystems), and RT-qPCR analysis was conducted using a ViiA^TM^7 System.

In addition, 50 ng of total RNA was reverse-transcribed into cDNA using a TaqMan MicroRNA Reverse Transcription kit (Applied Biosystems). This was followed by a miRNA expression analysis using a TaqMan Fast Advanced Master Mix (Applied Biosystems). The relative quantification of the expression levels was conducted using the 2^−ΔΔCT^ method.

## 5. Conclusions

In this study, alterations in gene expressions across CSCs and the EMT were identified, suggesting the presence of cross-talk between these two processes. Furthermore, this study focused on common, as well as specific gene and miRNA markers that are correlated with overall survival rates, and these are summarized and presented in Figure 7. It is apparent that the EMT mechanism as regulated by CSC stemness features are linked, thus revealing different therapeutic vulnerabilities, and clearing the way for novel cancer treatments.

Overall, this study supports the utility of public databases to investigate molecular mechanisms involved in BRCA. In particular, this study provided detailed knowledge of molecular mechanisms associated with EMT and CSCs, and identified markers useful for survival outcome predictions. This knowledge can also be useful in identifying novel cancer drugs and for pursuing associated research studies.

## Figures and Tables

**Table 1:** Top 10 upregulated and downregulated genes and miRNAs.

Gene Expression	Gene	*p*-Value	Log2 (Fold Change)	FDR (*p*-Value)	miRNA Expression Level	Symbol	*FDR (p*-Value)	Log2 (Fold Change)	FDR (*p*-Value)
Upregulated genes	COL10A1	2.40 × 10^−176^	6.76	5.47 × 10^−173^	Upregulated miRNAs	hsa-mir-183	4.01 × 10^−92^	2.95	1.40 × 10^−89^
	COL11A1	3.75 × 10^−101^	5.88	2.81 × 10^−99^		hsa-mir-96	3.59 × 10^−90^	2.87	9.38 × 10^−88^
	MMP11	1.27 × 10^−176^	5.82	3.27 × 10^−173^		hsa-mir-141	2.12 × 10^−76^	2.71	3.16 × 10^−74^
	MMP13	6.76 × 10^−94^	5.79	3.94 × 10^−92^		hsa-mir-429	4.08 × 10^−63^	2.68	3.88 × 10^−61^
	CST1	1.22 × 10^−65^	5.64	2.52 × 10^−64^		hsa-mir-200a	2.21 × 10^−60^	2.58	1.93 × 10^−58^
	MMP1	1.43 × 10^−61^	4.95	2.58 × 10^−60^		hsa-mir-196a-1	2.43 × 10^−19^	2.41	2.86 × 10^−18^
	PPAPDC1A	9.77 × 10^−121^	4.87	1.50 × 10^−118^		hsa-mir-182	4.55 × 10^−72^	2.36	5.29 × 10^−70^
	COMP	2.45 × 10^−90^	4.76	1.27 × 10^−88^		hsa-mir-210	2.78 × 10^−22^	2.36	3.87 × 10^−21^
	NEK2	8.02 × 10^−196^	4.59	8.23 × 10^−192^		hsa-mir-21	9.49 × 10^−139^	2.32	9.93 × 10^−136^
	PKMYT1	9.28 × 10^−174^	4.56	1.73 × 10^−170^		hsa-mir-190b	1.14 × 10^−31^	2.31	2.34 × 10^−30^
Downregulated genes	ITSN1	5.46 × 10^−89^	−1.5	2.61 × 10^−87^	Downregulated miRNAs	hsa-mir-378c	2.06 × 10 -47	−1.51	9.00 × 10^−46^
	C3orf64	2.19 × 10^−88^	−1.5	1.02 × 10^−86^		hsa-mir-675	2.29 × 10^−17^	−1.52	2.40 × 10^−16^
	C2CD2	1.62 × 10^−60^	−1.5	2.85 × 10^−59^		hsa-mir-1258	1.54 × 10^−58^	−1.56	1.24 × 10^−56^
	CAB39L	4.35 × 10^−49^	−1.5	5.34 × 10^−48^		hsa-mir-1-2	2.60 × 10^−32^	−1.60	5.44 × 10^−31^
	SLC5A4	1.08 × 10^−44^	−1.5	1.13 × 10^−43^		hsa-mir-205	8.64 × 10^−10^	−1.71	4.97 × 10^−09^
	KALRN	3.50 × 10^−40^	−1.5	3.09 × 10^−39^		hsa-mir-584	2.52 × 10^−33^	−1.71	5.49 × 10^−32^
	FZD7	2.30 × 10^−37^	−1.5	1.85 × 10^−36^		hsa-mir-511-1	1.39 × 10^−40^	−1.75	4.55 × 10^−39^
	TMIE	2.38 × 10^−37^	−1.5	1.92 × 10^−36^		hsa-mir-483	2.22 × 10^−32^	−1.78	4.74 × 10^−31^
	NCOA7	3.07 × 10^−37^	−1.5	2.46 × 10^−36^		hsa-mir-125b-2	5.03 × 10^−44^	−1.79	1.95 × 10^−42^
	HAPLN4	4.90 × 10^−29^	−1.5	2.87 × 10^−28^		hsa-mir-511-2	7.51 × 10^−45^	−1.82	3.02 × 10^−43^

**Table 2:** Clinical characteristics of breast patients included in this study (T: tumor, N: lymph nodes, and M: metastasis). ER: estrogen receptor, PR: progesterone receptor, and HER2: human epidermal growth factor receptor 2.

No.	Age	Diagnostic	T	N	M	Stage	ER	PR	HER2/New
1	57	invasive ductal carcinomas	2	0	X	IIB	+	+	−
2	68	invasive ductal carcinomas	1C	1	X	IIA	+	+	−
3	42	invasive ductal carcinomas	1a	1a	X	I	+	+	+
4	56	invasive ductal carcinomas	2	0	0	IIB	+	+	−
5	70	invasive ductal carcinomas	2	0	0	IIa	+	+	−
6	43	invasive ductal carcinomas	2	1	0	IIB	+	+	+
7	73	invasive ductal carcinomas	2	0	0	IIA	+	+	+
8	76	invasive ductal carcinomas	2	1	X	IIB	+	+	−
9	66	invasive ductal carcinomas	2	1	0	IIB	+	+	−
10	67	invasive ductal carcinomas	2	0	0	IIA	+	+	−
11	62	invasive ductal carcinomas	2	1	0	IIB	−	−	+
12	33	invasive ductal carcinomas	1C	0	0	IA	+	+	−
13	62	invasive ductal carcinomas	2	0	0	IIA	+	+	−
14	67	invasive ductal carcinomas	1C	1	0	IIB	+	+	−
15	39	invasive ductal carcinomas	2	0	X	IIA	+	+	+
16	53	invasive ductal carcinomas	1c	0	0	I	+	+	−
17	59	invasive ductal carcinomas	2	0	0	IIA	+	+	−
18	75	invasive ductal carcinomas	2	1	0	IIB	+	+	−
19	64	invasive ductal carcinomas	4	1	0	IIIA	+	−	−
20	41	invasive ductal carcinomas	1c	1a	0	IIA	+	+	−
21	33	invasive ductal carcinomas	2	1	0	IIB	+	+	+
22	73	invasive ductal carcinomas	1c	1	X	IIB	+	+	+
23	56	invasive ductal carcinomas	2	0	0	IB	+	+	−
24	51	invasive ductal carcinomas	2	0	0	IB	+	+	−
25	64	invasive ductal carcinomas	2	0	0	IB	+	+	−
26	71	invasive ductal carcinomas	1c	0	0	IA	+	+	+
27	53	invasive ductal carcinomas	2	0	0	IB	+	+	+
28	38	invasive ductal carcinomas	1c	0	X	IIB	+	+	−
29	42	invasive ductal carcinomas	1c	1a	X	IA	+	+	−
30	35	invasive ductal carcinomas	2	0	0	IC	+	+	−

## Supplementary Materials

The following are available online at https://www.mdpi.com/2072-6694/12/10/3053/s1: Table S1. Altered gene expression signature in BRCA, Table S2. Altered miRNA signature in BRCA and Table S3. Main mutation related to BRCA.
